# Cross-Sectional Prevalence of SARS-CoV-2 Among Skilled Nursing Facility Employees and Residents Across Facilities in Seattle

**DOI:** 10.1007/s11606-020-06165-7

**Published:** 2020-09-01

**Authors:** Ana A. Weil, Kira L. Newman, Thuan D. Ong, Giana H. Davidson, Jennifer Logue, Elisabeth Brandstetter, Ariana Magedson, Dylan McDonald, Denise J. McCulloch, Santiago Neme, James Lewis, Jeff S. Duchin, Weizhi Zhong, Lea M. Starita, Trevor Bedford, Alison C. Roxby, Helen Y. Chu

**Affiliations:** 1 Department of Medicine, Division of Allergy and Infectious Diseases, University of Washington, Seattle, WA USA; 2 Department of Surgery, University of Washington, Seattle, WA USA; 3 Department of Health Services, University of Washington, Seattle, WA USA; 4 Public Health - Seattle & King County, King County, WA USA; 5 Department of Genome Sciences, University of Washington, Seattle, WA USA; 6 Brotman Baty Institute for Precision Medicine, Seattle, WA USA; 7 Fred Hutchinson Cancer Research Center, Seattle, WA USA; 8 Department of Global Health, University of Washington, Seattle, WA USA

**Keywords:** SARS-CoV-2, skilled nursing facility

## Abstract

**Background:**

Skilled nursing facilities (SNFs) are high-risk settings for SARS-CoV-2 transmission. Infection rates among employees are infrequently described.

**Objective:**

To describe SARS-CoV-2 rates among SNF employees and residents during a non-outbreak time period, we measured cross-sectional SARS-CoV-2 prevalence across multiple sites in the Seattle area.

**Design:**

SARS-CoV-2 testing was performed for SNF employees and residents using quantitative real-time reverse transcription polymerase chain reaction. A subset of employees completed a sociodemographic and symptom questionnaire.

**Participants:**

Between March 29 and May 13, 2020, we tested 1583 employees and 1208 residents at 16 SNFs for SARS-CoV-2.

**Main Measure:**

SARS-CoV-2 testing results and symptom report among employees and residents.

**Key Results:**

Eleven of the 16 SNFs had one or more resident or employee test positive. Overall, 46 (2.9%) employees had positive or inconclusive testing for SARS-CoV-2, and among those who completed surveys, most were asymptomatic and involved in direct patient care. The majority of employees tested were female (934, 73%), and most employees were Asian (392, 30%), Black (360, 28%), or white (360, 28%). Among the 1208 residents tested, 110 (9.1%) had positive or inconclusive results. There was no association between the presence of positive residents and positive employees within a SNF (*p* = 0.62, McNemar’s test).

**Conclusions:**

In the largest study of SNFs to date, SARS-CoV-2 infections were detected among both employees and residents. Employees testing positive were often asymptomatic and involved in direct patient care. Surveillance testing is needed for SNF employees and residents during the pandemic response.

**Electronic supplementary material:**

The online version of this article (10.1007/s11606-020-06165-7) contains supplementary material, which is available to authorized users.

## INTRODUCTION

Skilled nursing facilities (SNFs) are high-risk settings for rapid spread of SARS-CoV-2 infection because they are congregate settings that frequently house a vulnerable patient population with multiple co-morbidities.^1, 2^ Many outbreaks in long-term care facilities have been described, often with high mortality rates in residents.^3^ Symptom screening alone for COVID-19 has been shown to be inadequate for preventing outbreaks in congregate settings, likely because of asymptomatic and presymptomatic spread.^4^ For this reason, the Centers for Medicaid and Medicare Services released guidance recommending baseline screening for SARS-CoV-2 in congregate settings and periodic screening of employees and residents.^5^ Given the essential role of SNF employees and their potential role in introducing SARS-CoV-2 into a high-risk setting, the prevalence of SARS-CoV-2 in SNF personnel is key to understanding outbreaks and disease transmission within SNFs.

The SNF care environment and SNF employees have specific characteristics that increase the risk of SARS-CoV-2 outbreaks in SNFs. The COVID-19 pandemic has accentuated realities of the SNF care environment in which resources and personnel are often inadequate to meet the demands of an infectious disease outbreak, and SNFs have also reported employee and supply shortages since the COVID-19 pandemic began.^6^ SNF employees also frequently work at multiple sites, experience higher turnover, receive lower pay compared with acute care settings, have less access to SARS-CoV-2 testing, and have been less prioritized to receive personal protective equipment (PPE) allocations.^7–11^ A recent account in our state describes SNF employees demonstrating heterogeneous PPE use and training, in addition to inadequate PPE supply and delayed recognition of cases.^12^ Due to fear of infection and/or lack of PPE, absenteeism in the SNF environment has also been reported.^13, 14^ Prior to the COVID-19 era, high levels of absenteeism in nursing facilities have been associated with poor outcomes.^15^

In Washington State, a government call to action on March 10, 2020, led to SNFs barring visitors and engaging in employee symptom-based screening.^16^ Despite these precautions, cases of SARS-CoV-2 continue to be observed in SNFs. To address this, testing for SARS-CoV-2 among SNF employees regardless of symptoms is needed in order to develop strategies for decreasing transmission in SNFs and the larger community. In this study, we describe the results of cross-sectional resident and employee SARS-CoV-2 testing, and infection control and personnel policies associated with 16 Seattle area SNFs.

## METHODS

Through two testing strategies, a total of 16 SNFs offered testing to either residents, employees, or both. The first testing strategy was directed by Public Health of Seattle & King County (PHSKC) and focused on SNF resident testing with employee testing offered at select sites. The second testing strategy was facilitated by the Seattle Flu Study (SFS) and directed at testing only employees. PHSKC testing was conducted by providers from University of Washington, between March 29, 2020, and May 8, 2020, at 13 SNFs and one assisted living facility, of which six offered both resident and employee testing and eight had only resident testing. Employee testing by SFS was designed to coincide with resident testing done by PHSKC when possible. SFS testing was conducted between April 14, 2020, and May 13, 2020, at 13 SNFs. At three SNFs, both the PHSKC and Seattle Flu Study teams tested SNF employees.

### Population

PHSKC identified SNFs in need of SARS-CoV-2 testing, including sites with known COVID-19 cases, facilities with no known cases, or where COVID-19 testing of residents had not occurred. For testing through PHSKC, teams of healthcare workers collected nasopharyngeal (NP) swabs from all residents in a SNF during a single visit. For testing through Seattle Flu Study, facilities identified by PHSKC were contacted by the study team for employee testing. Facilities agreeing to participate messaged all employees before the visit to inform them of the upcoming testing event and distributed a copy of the informed consent form for previewing. Employees were eligible to participate if they worked at the facility and were over 18 years old. All testing was voluntary and not required by the employer, and employees were advised that results would not be reported directly to employers. Employees who reported prior testing for SARS-CoV-2 through other mechanisms were eligible for enrollment. Study staff consented individuals in English or in the participant’s language of preference using an interpreter. After informed consent was obtained, individuals completed an electronic tablet–based questionnaire (Project Redcap in REDCap, Nashville, TN) and self-collected a mid-nasal swab under observation by trained study staff.

### Laboratory Methods

For testing through PHSKC, NP swabs from SNF residents were placed in universal viral transport media (Becton Dickinson, Franklin, NJ) and transported to the University of Washington Virology Laboratory for testing via a one-step real-time reverse transcription polymerase chain reaction (RT-PCR) assay following the SARS-CoV-2 CDC assay protocol, as previously described.^17^ No samples tested through PHSKC were resulted as indeterminant.

For testing through Seattle Flu Study, self-collected mid-nasal nylon-flocked swabs were placed in universal viral transport media (Becton Dickinson, Franklin, NJ) and transported to the Brotman Baty Institute for Precision Medicine and the Northwest Genomics Center for testing using a laboratory-developed test for SARS-CoV-2, as previously described.^18^ Briefly, SARS-CoV-2 detection was performed using real-time RT-PCR with a probe set targeting Orf1b and S with FAM fluor (Life Technologies 4332079 assays # APGZJKF and APXGVC4APX) multiplexed with an RNase P probe set with VIC or HEX fluor (Life Technologies A30064 or Integrated Data Technologies custom made) each in duplicate on a QuantStudio 6 instrument (Applied Biosystems). Three or four replicates for RNase P and SARS-CoV-2 were required to have a detection cycle threshold less than 40 for a sample to be considered positive for this laboratory-developed test, or both replicates must be positive in the research assay. Samples resulting with two replicates of positive SARS-CoV-2 detection were defined as inconclusive. Because tests determined to be inconclusive had SARS-CoV-2 detected in multiple replicates, these results were grouped with positive results for reporting purposes.

### Data Collection

For individuals tested through PHSKC, data available included name, date of birth, date of testing, and whether the individual was a resident or an employee. For employees tested through the Seattle Flu Study, data included participant date of birth, date of testing, race and ethnicity, location and nature of work, new symptoms experienced during the last 7 days, and history of SARS-CoV-2 testing (Appendix 1 in the Supplementary Material). Information on SNF policies regarding absenteeism, infection control, and employee health were collected from SNF management by email 2 weeks following employee testing using a standardized data collection form (Appendix 2 in the Supplementary Material).

### Data Analysis

Time between resident and employee testing was calculated as the days elapsed between first testing dates for each group at a SNF. For sites with multiple testing dates for employees, residents, or both, tests from all dates for a given group were combined to calculate the prevalence at each site. All data analysis was conducted in the R statistical language (R Foundation for Statistical Computing, Vienna, Austria). Frequencies were tabulated for social and demographic data. To test the association between residents and employees who tested positive for SARS-CoV-2, a two-tailed McNemar’s test was used. *p* values < 0.05 were considered statistically significant.

### Reporting

For employees, positive or inconclusive SARS-CoV-2 test results were reported directly to participants by phone within 48 h and to the Washington State Department of Health. Resident results were reported to the ordering physician at the SNF.

### Ethics

The Seattle Flu Study was approved by the University of Washington Institutional Review Board. Other testing of residents and employees was conducted as a public health surveillance activity under the direction of PHSKC.

## RESULTS

### Employee Testing Results

**Figure 1:**
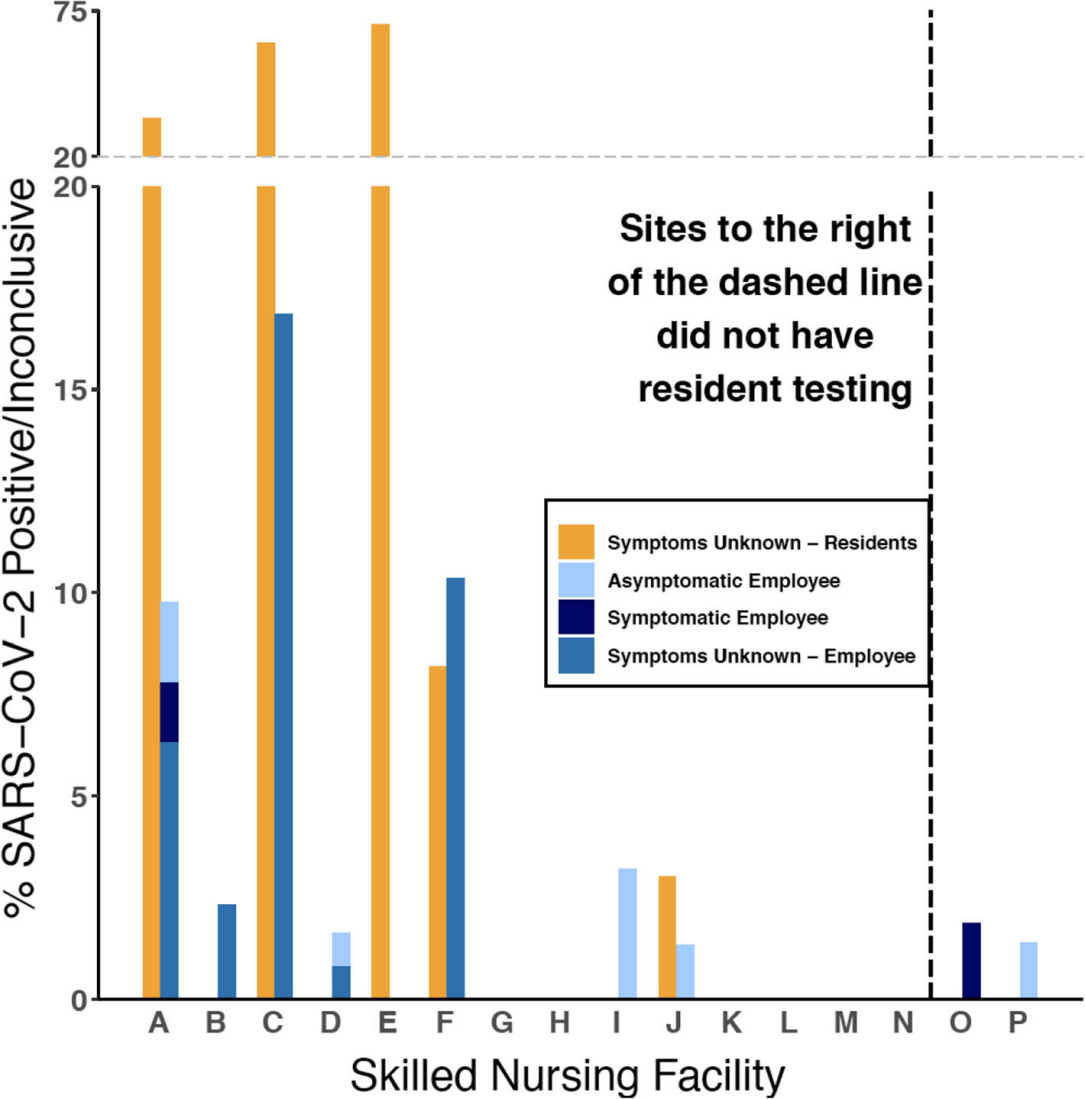
**Proportion of positive or inconclusive test results for SARS-CoV-2 at skilled nursing facilities, stratified by residents and employees, and self-reported symptoms. Letters represent individual skilled nursing facilities. Symptomatic study participants reported at least one of the following new symptoms at time of sample collection: fever, headache, cough, chills, sweats, sore throat, nausea or vomiting, rhinorrhea, fatigue, myalgia, dyspnea, diarrhea, anosmia, or ageusia.**

Overall, 1583 employees at 16 SNFs were tested, with 287 (18%) tested through PHSKC and 1296 (82%) through SFS. A total of 46 (2.9%) employees had positive or inconclusive testing for SARS-CoV-2 (Fig. 1; Supplemental Table 1). Demographic information from employees tested through SFS is shown in Table 1. This information is not available for employees tested through PHSKC. The majority of employees tested were female (934, 72%) and identified their race as Asian (392, 30%), Black (360, 28%), or white (360, 28%), and worked in direct patient care (795, 62%). Only 8.1% (105) of employees reported working at more than one SNF. New symptoms were reported in 8.7% (106) during the week prior to testing. Most employees (930, 73%) had not previously been tested for SARS-CoV-2. Of employees who reported prior testing, 13% (46) had a previous positive or inconclusive test result.

**Table 1:** Demographics and Clinical Characteristics of Skilled Nursing Facility Employees Tested Through Seattle Flu Study Stratified by SARS-CoV-2 Test Result

	Total, *N* = 1289	Positive/inconclusive, *N* = 13	Negative, *N* = 1276
Mean age (years), [range]	45 [18, 83]	50 [18, 69]	45 [18, 83]
Female sex (%)	934 (73)	10 (77)	924 (73)
Race			
American Indian/Alaska Native	3 (0.2)	0 (0.0)	3 (0.2)
Asian	392 (30)	4 (31)	388 (30)
Black	360 (28)	3 (23)	357 (28)
Native Hawaiian/Pacific Islander	34 (2.6)	0 (0)	34 (2.7)
Other/multiple	111 (8.6)	2 (15)	109 (8.5)
White	360 (28)	1 (7.7)	359 (28)
Hispanic/Latinx ethnicity	109 (8.5)	3 (23)	106 (8.3)
Work type at enrollment facility			
Direct patient care	795 (62)	10 (77)	785 (62)
Other*	473 (37)	3 (23)	470 (37)
Employed at multiple SNFs	105 (8.2)	1 (7.7)	104 (8.2)
Symptoms			
None	1179 (92)	9 (69)	1170 (92)
Cough	22 (1.7)	2 (15)	20 (1.6)
Sore throat	39 (3.0)	1 (7.7)	38 (3.0)
Fever	7 (0.5)	0 (0.0)	7 (0.5)
COVID-like illness^†^	2 (0.2)	0 (0.0)	2 (0.2)
Other respiratory symptoms^†^	40 (3.1)	0 (0.0)	40 (3.1)
Gastrointestinal symptoms^†^	12 (0.9)	2 (15)	10 (0.8)
Other systemic symptoms^†^	30 (2.3)	0 (0.0)	30 (2.4)
Prior SARS-CoV-2 test			
Positive/inconclusive	46 (3.6)	6 (46)	40 (3.1)
Negative	309 (24.0)	3 (23.1)	306 (24.1)
None	930 (72)	4 (31)	926 (73)
Days since prior test; median [IQR]	17 [10, 34]	17 [10, 17]	17 [11, 35]
Days of work missed; median [IQR]	0 [0, 7]	7.5 [6, 8]	0 [0, 5]

Percentages may not sum to 100% due to missing responses

Other respiratory symptoms include dyspnea, rhinorrhea, anosmia, and ageusia

Gastrointestinal symptoms are defined as diarrhea, nausea, or vomiting

Other systemic symptoms include chills, fatigue, myalgia, or sweats

*Non-patient care positions include administration, facilities, food service, and transportation

†COVID-19-like illness is defined as fever and cough or shortness of breath

Of the 46 total employees who tested positive for SARS-CoV-2, 33 were tested through the PHSKC and 13 through SFS, and only the latter 13 had accompanying survey data. The majority of those tested through SFS reported performing direct patient care (Table 1). Employees who tested positive were more likely to have had a prior positive test compared with employees that tested negative (6 (46%) versus 40 (3%), respectively). Employees who tested positive were less likely to be asymptomatic (9 (69%) versus 1170 (92%), respectively). Among the six individuals with SARS-CoV-2 detected who had not previously been tested, only one (14%) reported symptoms.

Based on the employee counts provided by SNFs, an average of 70% (range 34–108%) of employees on-site on the day of testing participated (Supplemental Table 2). Despite facility policies that any employees with new respiratory symptoms should not come to work, several employees reported respiratory symptoms (2 with cough, 1 with sore throat) and tested positive in our study, and several employees reporting gastrointestinal symptoms also tested positive.

### Resident Testing Results

Residents of 14 SNFs were tested through the PHSKC. Of 1208 residents, 110 (9.1%) tested positive for SARS-CoV-2 (Fig. 1; Supplemental Table 1). Resident testing was conducted within an average of 1.6 days (range 0–7 days) of employee testing. Five SNFs (36%) had SARS-CoV-2-positive residents, and among SNFs with at least one resident who tested positive, the mean positivity rate for SARS-CoV-2 was 33% (range, 3.0–70%).

### Relationship Between Employees and Resident Testing Results

Of the 14 SNFs with resident and employee testing (Fig. 1), four (29%) had both positive residents and positive employees, three (22%) had positive employees but no positive residents, and one (7.1%) had positive residents but no positive employees. There was no significant association between presence of positive residents and positive employees (*p* = 0.62).

### Facility Policies

Thirteen of the 16 SNFs responded to the survey (Supplemental Table 2), although only six sites completed all survey questions. All sites reported that they had a policy in place that employees with any new respiratory symptoms should not come to work, and all sites reported having paid sick leave policies. These policies varied with regard to eligibility and how leave was made available. For example, one SNF reported a paid sick leave policy that allowed employees to accrue negative sick leave if necessary, while others did not allow this option. Most SNFs reported following the Centers for Disease Control guidelines for return to work after a respiratory illness.^19^ Seven of the 16 SNFs reported their PPE policy and all required universal masking. The type of mask used was not specified.

## DISCUSSION

We report the results of a large cross-sectional study evaluating SARS-CoV-2 prevalence in skilled nursing facilities (SNFs) in the Seattle area during the spring 2020 peak of the COVID-19 pandemic. To our knowledge, this is the first study to report occupational SARS-CoV-2 for SNF workers outside of an outbreak investigation and the largest study to date evaluating prevalence of SARS-CoV-2 in SNF workers.

We detected SARS-CoV-2 in both residents and employees at multiple sites. The majority of employee testing positive for SARS-CoV-2 were asymptomatic and involved in direct patient care. In this study, all surveillance was performed starting at least 2.5 weeks after the implementation of a strict no visitor policy at SNFs throughout the region. Given that many infected SNF employees were asymptomatic, transmission events have the potential to go undetected without broad-based testing of all employees. This is critical, because asymptomatic and presymptomatic infections have led to significant transmission events in other high-density congregate living facilities with high-risk residents.^1, 4^ Similar to other congregate environments like cruise ships, correctional institutions, and long-term care facilities,^20–22^ SNFs are an environment where introduction of one case may lead to rapid transmission. We did not assess for routes of disease transmission during the study period, and transmission of SARS-CoV-2 to SNF residents could have occurred from contact between residents, infected employees, or other outside contacts, such as exposure to dialysis centers outside of the SNF.

Minority communities are over-represented in low-wage healthcare clinics and include populations known to be disproportionately impacted by COVID-19.^9, 23–25^ We found that SNF employees in our study were disproportionately more non-white and non-Hispanic individuals, including a higher proportion of Asian and Black participants, than are represented in the population of the Seattle area.^26^ While all facilities reported some form of paid sick leave program, many had limits on eligibility for part-time and contracted employees. Lacking paid sick leave is a financial disincentive to report symptoms or positive tests. These factors highlight the vulnerability of SNF employees as an often overlooked group in the transmission of SARS-CoV-2.

This study was conducted during an ongoing pandemic in Seattle. In the conduct of this study, we encountered obstacles including shortages of PPE, viral transport media, and nasal swabs. Participation was voluntary across sites, and an average of 70% on-site employees participated. The administration at each SNF presented the opportunity for testing with different levels of enthusiasm and support, and this may have impacted employee participation. Furthermore, when approached with the offer of employee testing, the administrative leadership of some of the SNFs in the Seattle area declined to participate. Common concerns about mass employee testing from SNF administration were that testing would result in increased fear, employee absenteeism, and/or consequent staffing shortages.

Strengths of this study include broad testing of both residents and employees in a group of SNFs at the spring 2020 peak of the COVID-19 pandemic in a major metropolitan area. We collected sociodemographic and symptom data on the majority of the employees, and information on infection control policies across sites. Limitations of this study included that sites were included only if administrative leadership agreed to participate, and the sites that did not participate may have differed in infection rates and PPE practices compared with those that agreed. Testing at sites did not include all employees; only employees volunteering for testing participated. Collection of SARS-CoV-2 samples used different collection methods between residents and employees, and the timing of testing for resident and employees was not simultaneous. However, both methods of collection have proven to be concordant (Citation of a manuscript under review will be inserted here) and the mean difference between employee and resident testing was small (mean of 1.6 days). Symptom data was self-reported and may be limited by a social desirability bias and/or by recall bias. To mitigate bias, employees filled out the questionnaire using an electronic tablet while at a six foot distance from other participants, which afforded some privacy. Additionally, we do not have longitudinal data on participants and do not know how many asymptomatic individuals were presymptomatic.

As SARS-CoV-2 infections continue to cause disproportionate numbers of deaths in facilities for older adults throughout the country, strategies to prevent mortality in this fragile population are critical. We found that infections in both employees and residents persisted even with no visitor policies, and facilities had heterogenous paid leave policies. Based on our findings, implementation of periodic point prevalence testing of both residents and employees, coupled with rigorous infection control precautions and universal paid sick leave for employees, may provide an improved strategy to reduce mortality in this highly vulnerable population. Future research should focus on trials of strategies, such as routine employee testing, to understand their effectiveness in SARS-CoV-2 high-risk occupational settings.

## Electronic Supplementary Material

ESM 1 (DOCX 22 kb)

ESM 2 (DOCX 20.3 kb)
