# Supplementary material for: Cross-Sectional Prevalence of SARS-CoV-2 Among Skilled Nursing Facility Employees and Residents Across Facilities in Seattle
Source: J Gen Intern Med. 2020 Sep 1;35(11):3302–7. doi: 10.1007/s11606-020-06165-7 (PMC7462112; doi:10.1007/s11606-020-06165-7)
Supplement: Supplementary file 1 — (DOCX 22 kb) [file 11606_2020_6165_MOESM1_ESM.docx]

**APPENDIX 1**

**Title:** Seattle Flu Study survey questionnaire for Skilled Nursing Facility Employees

| date | Date of Enrollment (Today's Date) | text (date_mdy) Field Annotation: @TODAY @HIDDEN |
| --- | --- | --- |
| address | Home Address - Street Name and House or Apartment Number | text, Identifier |
| city | City or Town | text, Required, Identifier |
| zipcode | Zip Code | text (zipcode), Required, Identifier |
| worksite | What facilities do you regularly work at? Select all that apply. | checkbox, Required   \| 1 \| worksite___1 \| \| --- \| --- \| \| 2 \| worksite___2 \| \| 3 \| worksite___3 \| \| 4 \| worksite___4 \| \| 5 \| worksite___5 \| \| 6 \| worksite___6 \| \| 7 \| worksite___7 \| \| 8 \| worksite___8 \| \| 9 \| worksite___9 \| \| 10 \| worksite___10 \| \| 11 \| worksite___11 \| \| 12 \| worksite___12 \| \| 13 \| worksite___13 \| \| 14 \| worksite___14 \| \| 15 \| worksite___15 \| \| 16 \| worksite___16 \| |
| worktype | What type of work do you do at this facility? | radio, Required   \| 1 \| Direct patient care \| \| --- \| --- \| \| 2 \| Facilities \| \| 3 \| Administrative \| \| 4 \| Food preparation \| \| 5 \| Transportation \| \| 6 \| Other \| |
| symptom_header_3 | Symptom Information | descriptive |
| symptoms | Which new or worsening symptoms have you experienced in the last 7 days? Select all that apply. | checkbox, Required   \| 1 \| Feeling feverish \| \| --- \| --- \| \| 2 \| Headache \| \| 3 \| Cough \| \| 4 \| Chills or shivering \| \| 5 \| Sweats \| \| 6 \| Sore throat or itchy/scratchy throat \| \| 7 \| Nausea or vomiting \| \| 8 \| Runny or stuffy nose \| \| 9 \| Feeling more tired than usual \| \| 10 \| Muscle or body aches \| \| 11 \| Increased trouble with breathing \| \| 12 \| Diarrhea \| \| 20 \| Unable to smell \| \| 21 \| Unable to taste \| \| 000 \| None of the above \|   Field Annotation: @NONEOFTHEABOVE = '000' |
| symptom_duration  Show the field ONLY if:  [symptoms(1)]="1" or [symptoms(2)]="1" or [symptoms(3)]="1" or [symptoms(4)]="1" or [symptoms(5)]="1" or [symptoms(6)]="1" or [symptoms(7)]="1" or [symptoms(8)]="1" or [symptoms(9)]="1" or [symptoms(10)]="1" or [symptoms(11)]="1" or [symptoms(12)]="1" or [symptoms(20)]="1" or [symptoms(21)]="1" | What day did your current symptoms start?  Note: Today's date is highlighted in yellow on the calendar.  *MM-DD-YYYY* | text (date_mdy), Required Field Annotation: @HIDEBUTTON |
| priortest | Have you previously been tested for COVID-19? | radio, Required   \| 0 \| No \| \| --- \| --- \| \| 1 \| Yes \| |
| priortestresult  Show the field ONLY if:  [priortest] = "1" | What was the result of this previous test? | radio, Required   \| 0 \| Negative \| \| --- \| --- \| \| 1 \| Positive \| \| 2 \| Inconclusive \| \| 3 \| I haven't received my result yet \| |
| priortestdt  Show the field ONLY if:  [priortest] = "1" | What was the date of this test? | text (date_mdy) |
| birthday | What is your birthday? Select a date by clicking the calendar image below.  *Make sure to select the correct year.* | text (date_mdy), Required Field Annotation: @HIDEBUTTON |
| sex | What is your sex? | radio, Required   \| 1 \| Male \| \| --- \| --- \| \| 2 \| Female \| \| 3 \| Other (please specify) \| \| 999 \| Prefer not to say \| |
| other_sex  Show the field ONLY if:  [sex] = 3 | Other sex: | text (alpha_only) |
| hispanic | Are you Hispanic or Latino? | radio, Required   \| 1 \| Yes \| \| --- \| --- \| \| 0 \| No \| \| 999 \| Prefer not to say \| |
| race | How would you describe your race? Select all that apply. | checkbox, Required   \| 1 \| race___1 \| American Indian or Alaska Native \| \| --- \| --- \| --- \| \| 2 \| race___2 \| Asian \| \| 3 \| race___3 \| Native Hawaiian or other Pacific Islander \| \| 4 \| race___4 \| Black or African American \| \| 5 \| race___5 \| White \| \| 6 \| race___6 \| Other \| \| 999 \| race___999 \| Prefer not to say \|   Field Annotation: @NONEOFTHEABOVE=999 |
| collection_date | Specimen collection date | text (date_mdy) Field Annotation: @TODAY @HIDDEN |

**APPENDIX 2**

**Title:** Standardized Data Collection Form sent to Skilled Nursing Facilities (SNF)

1. Total number of staff at your SNF broken down by full time / part time / contracted?

2. Number working on the site on the day of testing?

3. Total number of residents at your site on the day of testing?

4. Nurse aide hours per resident on the day of testing?

5. Total number of licensed nurse staff hours per resident per day on the day of testing?

6. Physical therapist staff hour per resident per day on the day of testing?

7. What your PPE protocols for employees at your facility was at the time of Seattle Flu Study COVID testing, and now if that has changed?

8. What your return-to-work protocol for your facility at the time of Seattle Flu Study COVID testing, and now if that has changed?

9. Whether or not you have paid sick leave, who that would apply to, and a brief description?

10. Absenteeism data: Two weeks before & after the date of testing, broken down by positions and employment status listed below.

|  | 2 Weeks Before Date of Testing | 2 Weeks After Date of Testing |
| --- | --- | --- |
| Positions |  |  |
| Direct patient care |  |  |
| Administrative |  |  |
| Facilities |  |  |
| Food prep |  |  |
| Other |  |  |
| Employment status |  |  |
| Full time |  |  |
| Part time |  |  |
| Contracted |  |  |
