# Supplementary material for: Cross-Sectional Prevalence of SARS-CoV-2 Among Skilled Nursing Facility Employees and Residents Across Facilities in Seattle
Source: J Gen Intern Med. 2020 Sep 1;35(11):3302–7. doi: 10.1007/s11606-020-06165-7 (PMC7462112; doi:10.1007/s11606-020-06165-7)
Supplement: Supplementary file 2 — (DOCX 20.3 kb) [file 11606_2020_6165_MOESM2_ESM.docx]

**Supplementary Table 1: Site-specific results including testing by Seattle Flu Study (SFS) and Public Health Seattle King County (PHSKC), March-May 2020.**

| Facility ID | Employee results (positive test results/total tested, all SFS participants unless otherwise specified) | Employee testing date(s) | Resident results (positive test results/total tested, all PHSKC participants) | Resident testing date(s) | Notes |
| --- | --- | --- | --- | --- | --- |
| A | 20/205  (SFS=7/139 PHSKC=13/66) | 3/29, 4/5, 4/15 | 28/81 | 3/29, 4/5, 4/14 | SFS testing 4/15, PHSKC testing 3/29 and 4/5 (n=66) |
| B | 1/43 (all PHSKC) | 4/14 | 0/68 | 4/15 |  |
| C | 15/89  (SFS=0/35 PHSKC=15/54) | 4/15, 5/7 | 44/70 | 4/15 | SFS testing 5/7, PHSKC testing 4/15 |
| D | 2/122  (SFS=1/117; PHSKC= 1/5) | 4/16 | 0/99 | 4/14-4/16 | SFS and PHSKC testing 4/16 |
| E | 0/71 | 4/17, 5/1 | 28/40 | 4/16 |  |
| F* | 3/29 (all PHSKC) | 4/18 | 9/110 | 4/18 |  |
| G | 0/90 (all PHSKC) | 4/20 | 0/115 | 4/17/20 |  |
| H | 0/105 | 4/21 | 0/117 | 4/21, 5/8 |  |
| I | 2/62 | 4/22 | 0/61 | 4/21 |  |
| J | 1/74 | 4/23 | 1/33 | 4/23 |  |
| K | 0/87 | 4/24 | 0/89 | 4/23 |  |
| L | 0/101 | 4/28 | 0/90 | 4/24 |  |
| M | 0/263 | 4/29, 4/30 | 0/150 | 4/25 |  |
| N | 0/110 | 5/5 | 0/85 | 4/28 |  |
| O | 1/53 | 5/6 | -- | -- | no resident sampling |
| P | 1/72 | 5/13 | -- | -- | no resident sampling |

*Facility F is an assisted living facility rather than a SNF.

**Supplementary Table 2.** **Skilled nursing facility employee availability for testing and COVID19 related policies.** PPE, return to work, and paid sick leave policies as of May 2020. CDC=Centers for Disease Control and Prevention; DOH=Washington State Department of Health; n=number; PPE=Personal protective equipment, NR=Not reported.

| Site ID | Employees on site on day of testing (total employees) | Residents on site on day of testing | Percentage of all employees tested (n=number of employees tested)* | PPE policy | Return to work policy | Paid sick leave |
| --- | --- | --- | --- | --- | --- | --- |
| A | Missing (202) | 64 | 102% (205) | NR | NR | Full-time employees only |
| B | 68 (109) | 70 | 39% (43) | Universal masking | CDC guidelines** | Yes |
| C | NR | NR | (89) | NR | NR | NR |
| D | 100 (130) | 107 | 94% (122) | Universal masking | Symptom free 72 hours. If tested, no return until negative result or if prior positive must have two negative results and 72 hours symptom-free. | Yes |
| E | 80 (151)† | 73 | 47% (71) | NR | NR | Yes |
| F | NR | NR | (29) | NR | NR | NR |
| G | 74 (146) | 117 | 62% (90) | NR | CDC guidelines | Yes |
| H | 82 (136) | 92 | 77% (105) | NR | NR | Yes |
| I | 59 (92) | 64 | 67% (62) | Universal masking | CDC guidelines | Yes |
| J | 52 (102) | 39 | 73% (74) | Universal masking | NR | Yes |
| K | NR | NR | (87) | NR | NR | NR |
| L | 90 (161) | 95 | 63% (101) | Universal masking for employees with direct resident care. Cloth masks for residents and employees with indirect care. | CDC guidelines | Yes |
| M | 228 (412) | 168 | 64% (263) | Universal masking; all new admits on droplet/contact for 7 days | If SARS-CoV-2-positive: symptom-free and 2 negative tests >24 hours apart after 2 week quarantine starting at first positive result.  SARS-CoV-2-suspected: removed from schedule until tested.  SARS-CoV-2-negative: until symptom-free for 72 hours | Full-time employees only |
| N | 60 (102) | 87 | 108% (110) | NR | NR | Yes |
| O | 84 (158) | 107 | 34% (53) | NR | NR | Yes |
| P | 68 (87) | 38 | 83% (72) | Universal masking | Washington State Dept of Health guidelines | Yes |

*Some percentages exceed 100 because employees were tested multiple times.

**Whether the symptom or testing-based CDC guidelines strategy was used in each case was not specified.

†Results are an average of two testing days.
